# Effectiveness of probiotics on the duration of illness in healthy children and adults who develop common acute respiratory infectious conditions: a systematic review and meta-analysis

**DOI:** 10.1017/S0007114514000075

**Published:** 2014-04-29

**Authors:** Sarah King, Julie Glanville, Mary Ellen Sanders, Anita Fitzgerald, Danielle Varley

**Affiliations:** 1 York Health Economics Consortium, York, UK; 2 Dairy and Food Culture Technologies, Centennial, CO80122, USA

**Keywords:** Probiotics, Respiratory infections, Systematic reviews

## Abstract

Recent systematic reviews have reported a positive, although modest, effect of probiotics in terms of preventing common cold symptoms. In this systematic review, the effect of probiotics, specifically *Lactobacillus* and *Bifidobacterium* strains, on the duration of acute respiratory infections in otherwise healthy children and adults was evaluated. To identify relevant trials, eight databases, including MEDLINE, Embase, the Cochrane Database of Systematic Reviews (CDSR), the Cochrane Central Register of Controlled Trials (CENTRAL), the Database of Abstracts of Reviews of Effects (DARE), Health Technology Assessment (HTA), Science Citation Index (SCI) and OAISTER, were searched from inception to 20 July 2012. Details regarding unpublished studies/databases were also obtained from probiotic manufacturers. Study selection, data extraction and quality assessment were carried out by two reviewers. Risk of bias was assessed using criteria adapted from those published by the Centre for Reviews and Dissemination. In this review, twenty randomised controlled trials (RCT) were included, of which twelve were considered to have a low risk of bias. Meta-analysis revealed significantly fewer numbers of days of illness per person (standardised mean difference (SMD) − 0·31 (95 % CI − 0·41, − 0·11), *I* ^2^= 3 %), shorter illness episodes by almost a day (weighted mean difference − 0·77 (95 % CI − 1·50, − 0·04), *I* ^2^= 80 %) (without an increase in the number of illness episodes), and fewer numbers of days absent from day care/school/work (SMD − 0·17 (95 % CI − 0·31, − 0·03), *I* ^2^= 67 %) in participants who received a probiotic intervention than in those who had taken a placebo. Reasons for heterogeneity between the studies were explored in subgroup analysis, but could not be explained, suggesting that the effect sizes found may differ between the population groups. This systematic review provides evidence from a number of good-quality RCT that probiotics reduce the duration of illness in otherwise healthy children and adults.

Respiratory infectious conditions place a substantial health and economic burden on society. In a 6-month survey of 3249 university students, upper respiratory tract infections (RTI) resulted in 6023 bed-days, 4263 missed school days, 3175 missed workdays and 45 219 d of illness^(^ ^1^ ^)^. According to the United States Centers for Disease Control and Prevention, twenty-two million school days and twenty million workdays in adults are lost annually due to the common cold in the USA^(^ ^2^ ^)^. The economic impact of colds has been estimated to be $40 billion dollars in the USA annually^(^ ^3^ ^)^.

Probiotics are defined as live micro-organisms that confer a health benefit on the host when administered in adequate amounts^(^ ^4^ ^)^. Several studies^(^ ^5^ ^–^ ^8^ ^)^ have evaluated the effectiveness of probiotics on the symptoms and incidence of common infectious respiratory diseases. Recent meta-analyses^(^ ^9^ ^,^ ^10^ ^)^ have reported a positive, although modest, effect of probiotics in terms of preventing common cold symptoms.

The objective of this systematic review was to assess the effect of probiotics on the duration of an acute RTI in otherwise healthy children and adults. We evaluated probiotics that belong to the *Lactobacillus* and *Bifidobacterium* genera, but not probiotics such as yeast and Gram-negative probiotics (e.g. Escherichia coli), which are taxonomically distinct. Thus, for the purposes of this review, ‘probiotic’ hereafter refers to *Lactobacillus* and *Bifidobacterium* strains.

## Methods

Studies eligible for inclusion in this systematic review were randomised controlled trials (RCT) of any duration that compared *Lactobacillus* and/or *Bifidobacterium* strains consumed orally with placebo or ‘no treatment’ in apparently healthy children (aged between 1 and 18 years) or adults who developed acute RTI at some point during the study. Open-label studies were eligible as long as the patients were randomised. The probiotic strains could be administered at any dose and could be combined with or not be combined with non-*Lactobacillus* or non-*Bifidobacterium* strains. Studies carried out using probiotics combined with other functional ingredients (such as prebiotics and vitamins) were also eligible as long as the comparator included the other functional ingredients, so that the overall effects could be attributed to the probiotic. To be eligible for inclusion, the trials had to report on a measure of illness duration, such as the length of illness episodes, number of days of illness per person, number of days off sick from day care, school or work, or time without an infection.

‘Acute respiratory infections’ were considered to include upper RTI and/or lower RTI, colds or influenza-like symptoms. Trials that reported on ‘common infectious disease’ were also included if RTI were defined within this umbrella term by the trial authors. Studies conducted in infants (aged < 1 year), trained athletes, seriously ill people and institutionalised elderly adults were excluded from this review as they were considered to be immunologically distinct.

To identify relevant trials, we searched a number of databases including MEDLINE, Embase, the Cochrane Database of Systematic Reviews (CDSR), the Cochrane Central Register of Controlled Trials (CENTRAL), the Database of Abstracts of Reviews of Effects (DARE), Health Technology Assessment (HTA) database, Science Citation Index (SCI) and OAISTER from inception to 20 July 2012. The search was not restricted by country or language. Search terms included (but were not limited to) ‘Probiotics’, ‘*Lactobacillus*’ and ‘*Bifidobacterium*’. The search results were combined with those obtained by searching for terms including (but not limited to) ‘Common Cold’, ‘Sinusitis’, ‘Pharyngitis’, ‘Laryngitis’ and ‘Respiratory Tract Infections’. The full set of search terms is presented in online Supplementary File S1.

Information on ongoing or recently completed trials, unpublished research and research reported in the grey literature was obtained by searching trial registers and selected major conference proceedings (3 years before search date). Unpublished studies were included in accordance with established approaches to the systematic review process^(^ ^11^ ^,^ ^12^ ^)^. The resources searched are also listed in online Supplementary File S1. The references of recent reviews and eligible studies were checked for additional trials not identified by the electronic search. Unpublished papers were also sought from the manufacturers within the funding organisation (Global Alliance for Probiotics), which included Yakult, Danone, Probi, Lallemand, Chr. Hansen, DuPont and Valio. Studies published only as abstracts or conference presentations were included if adequate data were provided.

An initial screening of search results was done by one reviewer to exclude obviously irrelevant records. The remaining records were screened by two reviewers independently to identify potentially relevant records meeting the inclusion/exclusion criteria. Full papers were obtained for these records and were assessed for relevance by two reviewers independently. Any discrepancies were resolved through discussion and/or by consulting a third reviewer.

For each eligible trial, data on study and population characteristics and results were extracted by one reviewer and checked by a second reviewer (Table 1). Quality assessment, using quality criteria adapted from the Centre for Reviews and Dissemination^(^ ^11^ ^)^, was also conducted by one reviewer and checked by a second reviewer (summaries are given in Table 2 and full assessments are provided in online Supplementary File S2). Any discrepancies in data extraction or quality assessment were resolved through discussion or by consulting a third reviewer. If any of the quality criteria were unclear in the trial publications, study authors were contacted for further (documented) information.

**Table 1:** Characteristics of the included studies

				Intervention	Comparator
Study	Country of study	Population group	Condition	Detail(s)	Number randomised (included in the analysis)	Details	Number randomised (included in the analysis)
Bentley (2008, unpublished results)	Germany	Adults at an increased risk of infection (at least two episodes in the previous 6 months)	Common cold	*Lactobacillus* *plantarum* HEAL9 and *L. paracasei* 8700:2 in maltodextrin	155 (146)	Placebo: sachet containing maltodextrin without living cultures	155 (138)
Berggren *et al.* ^(^ ^5^ ^)^	Sweden	Healthy adults aged 18–65 years	Common cold	*L. plantarum* HEAL9 and *L. paracasei* 8700:2 in maltodextrin	159 (137)	Placebo: sachet containing maltodextrin without living cultures	159 (135)
Cáceres *et al.* ^(^ ^23^ ^)^	Chile	Children (aged 1–5 years) attending day-care centres	Acute respiratory tract infections	*L. rhamnosus* HN001	203 (170)	Placebo: milk product with no probiotic	195 (179)
Cazzola *et al.* ^(^ ^20^ ^)^	France	Children (aged 3–7 years) attending school	Common winter diseases (URTI, LRTI and gastrointestinal tract infections)	*L. helveticus* R0052, *Bifidobacterium* *infantis* R0033 and *Bifidobacterium bifidum* R0071	52 (62)	Placebo: contained only common excipients	73 (50)
de Vrese *et al.* ^(^ ^6^ ^)^	Germany	Healthy adults (aged 18–67 years)	Common cold	*L.* *gasseri* PA 16/8, *Bifidobacterium* *longum* SP 07/3 and *B. bifidum* MF 20/5	238 (225)	Placebo: vitamin mineral preparation without probiotic	241 (229)
Guillemard *et al.* ^(^ ^24^ ^)^	Germany	Healthy shift workers	CID*	Actimel, *L. paracasei* subsp. *casei* DN-114001 combined with *Streptococcus thermophilus*+*L. delbrueckii* subsp. *bulgaricus*	500 (500)	Placebo: dairy drink without active components	500 (500)
Guillemard *et al.* ^(^ ^25^ ^)^	France	Elderly (not living in an institution)	URTI	Actimel, *L. paracasei* subsp. *casei* DN-114001 combined with *S. thermophilus*+*L. delbrueckii* subsp. *bulgaricus*	537 (535)	Placebo: dairy drink without active components	535 (535)
Hatakka (2007, unpublished results) and Hatakka *et al.* ^(^ ^18^ ^)^	Finland	Children (aged 1–6 years) attending day-care centres	Gastrointestinal and respiratory infections	*L. rhamnosus* GG	Unclear (282)	Placebo: milk without probiotic	Unclear (289)
Hojsak *et al.* ^(^ ^28^ ^)^	Croatia	Hospitalised children aged 12 months and older	Respiratory symptoms	*L. rhamnosus* GG	376 (376)	Placebo: fermented milk product without *L. rhamnosus* GG	366 (366)
Hojsak *et al.* ^(^ ^29^ ^)^	Croatia	Children attending day-care centres	Respiratory symptoms	*L. rhamnosus* GG	139 (139)	Placebo: fermented milk product without *L. rhamnosus* GG	142 (142)
Kloster *et al.* ^(^ ^27^ ^)^	Norway	Children (aged 12–36 months) attending day-care centres	Gastrointestinal and respiratory symptoms	Biola with *L. rhamnosus* GG, *L. acidophilus* LA-5 and *Bifidobacterium lactis* BB-12	117 (97)	Placebo: fermented milk drink without probiotic bacteria	123 (102)
Kumpu *et al.* ^(^ ^19^ ^)^	Finland	Children (aged 2–6 years) attending day-care centres	Respiratory symptoms	*L. rhamnosus* GG	261 (251)	Placebo: fresh milk without *L. rhamnosus*	262 (250)
Leyer *et al.* ^(^ ^7^ ^)^	China	Children (aged 3–5 years)	Cold and influenza-like symptoms	Two interventions:		Placebo: sucrose added to 1 % fat milk	104 (104)
				*L. acidophilus* NCFM (ATCC 700396)	110 (110)		
				*L. acidophilus* NCFM and *Bifidobacterium animalis* subsp. *lactis* Bi-07 (ATCC PTA-4802)	112 (112)		
Merenstein *et al.* ^(^ ^21^ ^)^	USA	Healthy children (aged 3–6 years) attending day care/school	CID*	DanActive, *L. paracasei* subsp. *casei* DN-114001 with *S. thermophilus* and *L. bulgaricus*	314 (250 households)	Placebo: non-fermented acidified dairy drink	324 (250 households)
Niborski (2008, unpublished results)	France	Healthy adults (mostly men) recruited from a fireman training course	CID*	*L. paracasei* subsp. *casei* DN-114001 with *S. thermophilus*+*L. delbrueckii* subsp. *bulgaricus*	118 (118)	Placebo: acidified milk (no bacteria)	121 (121)
Prodeus (2008, unpublished results)	Russia	Children (aged 3–5 years) attending day-care centres	URTI	*L. paracasei* subsp. *casei* DN-114001 with *S. thermophilus*+*L. delbrueckii* subsp. *bulgaricus*	300 (300)	Placebo: acidified milk (no bacteria)	299 (299)
Smith *et al.* ^(^ ^22^ ^)^	USA	Apparently healthy college students	URTI	*L. rhamnosus* GG and *B. lactis* BB-12	114 (101)	Placebo: powder without probiotic	117 (97)
Tiollier *et al.* ^(^ ^15^ ^)^	France	Male cadets undergoing commando training	Respiratory tract infections	*L. paracasei* subsp. *casei* DN-114001	24 (24)	Placebo: non-fermented milk	23 (23)
Tubelius *et al.* ^(^ ^16^ ^)^	Sweden	Healthy employees working at TetraPak	Respiratory and/or gastrointestinal tract infections	Probiotic drinking straw with *L. reuteri protectis*	132 (94)	Placebo: drinking straw without probiotic	130 (87)
Turchet *et al.* ^(^ ^17^ ^)^	Italy	Free-living elderly aged above 60 years	CID*	*L. casei* DN-114001	180 (180)	No treatment	180 (180)

URTI, upper respiratory tract infections; LRTI, lower respiratory tract infections.

* CID included URTI, LRTI and gastrointestinal tract infections.

**Table 2:** Quality criteria and study risk of bias assessment

Reference	Was randomisation carried out appropriately?	Was the concealment of treatment allocation adequate?	Were the care providers, participants and outcome assessors blind to treatment allocation?	Were the groups similar at the onset of the study in terms of prognostic factors, for example, severity of the disease?	Were there any unexpected imbalances in dropouts between the groups? If so, were they explained or adjusted for?	Is there any evidence to suggest that the authors measured more outcomes than they reported?	Did the analysis include an ITT analysis? If so, was this appropriate?
Bentley (2008, unpublished results)	L	L	L	L	L?	L	L
Berggren *et al.* ^(^ ^5^ ^)^	L	L	L	L	L	L	H
Cáceres *et al.* ^(^ ^23^ ^)^	L	L	L	L	H	Y	H
Cazzola *et al.* ^(^ ^20^ ^)^	L	L	L	L	L	L	L
de Vrese *et al.* ^(^ ^6^ ^)^	L	L	L	L	L	L	H
Guillemard *et al.* ^(^ ^24^ ^)^	L	L	L	L	L	L	L
Guillemard *et al.* ^(^ ^25^ ^)^	L	L	L	L	L	L	L
Hatakka (2007, unpublished results) and Hatakka *et al.* ^(^ ^18^ ^)^	L	L	L?	L?	L	L	H
Hojsak *et al.* ^(^ ^28^ ^)^	L	L	L	L	L	L	L
Hojsak *et al.* ^(^ ^29^ ^)^	L	L	L	L	L	L	L
Kloster *et al.* ^(^ ^27^ ^)^	L	L?	L?	L	L	L	H
Kumpu *et al.* ^(^ ^19^ ^)^	L	L	L?	L?	?	L	H
Leyer *et al.* ^(^ ^7^ ^)^	L	L	L?	L	L	L	L
Merenstein *et al.* ^(^ ^21^ ^)^	L	L	L	L	L?	L	L
Niborski *et al.* ^(^ ^14^ ^)^	L	L	L	L	L	L	L
Prodeus *et al.* ^(^ ^26^ ^)^	L	L	L	L?	L	L	L
Smith *et al.* ^(^ ^22^ ^)^	L	L	L	L	H	L	H
Tiollier *et al.* ^(^ ^15^ ^)^	L	L	L	L	L	L	L
Tubelius *et al.* ^(^ ^16^ ^)^	L	L	L	L	L	L	H
Turchet *et al.* ^(^ ^17^ ^)^	L	L?	H	L?	H	L	L

ITT, intention-to-treat; L, low risk; L?, low risk with some areas of uncertainty; H, high risk; ?, unclear risk.

Means and standard deviations were collected for continuous outcomes. In the absence of information, authors were contacted for further details. If no further data could be obtained, a standard deviation was imputed using *P* values or sd from other similar studies (where possible). For studies involving two probiotic treatment arms, the means and standard deviations from the two groups were combined using statistical software to create a single pairwise comparison with the placebo group. Where appropriate, data from all the studies were pooled in a meta-analysis to determine the overall effect size (weighted mean difference) with 95 % CI using a random-effects model. When the same outcome was measured in different ways, the data were pooled using a standardised mean difference (SMD) statistic. When continuous and dichotomous data were presented for an outcome, SMD (or log OR) and their standard errors were computed for all the studies to facilitate pooling of the data. Meta-analysis was conducted using RevMan software (The Nordic Cochrane Centre, The Cochrane Collaboration)^(^ ^13^ ^)^.

Heterogeneity across the studies was investigated using the χ^2^ test (significance set at *P*< 0·1) and the *I* ^2^ statistic (with a value ≥ 50 %) and by examining the random-effects between-study variance (τ^2^). When significant heterogeneity was evident, subgroup analysis planned *a priori* was used to explore differences among the trials for specific variables including age (children and adults), sex, country of study, treatment dose, intervention duration, and single-strain *v.* combination interventions. We also evaluated studies by their overall risk of bias (low, high or unclear) based on the Cochrane Risk of Bias tool. For the purposes of this review, a study was assumed to have a ‘low risk of bias’ when all the key quality criteria (i.e. randomisation method, allocation concealment and blinding) as well as most of the other criteria were adequately met; an ‘unclear risk’ of bias when most of the key criteria were not reported or unclear; and a ‘high risk’ of bias when one or more of the key criteria were not adequately met. The category ‘some risk of bias’ was assigned when all aspects of the key criteria were adequate, but either ^(^ ^1^ ^)^ an intention-to-treat analysis was not conducted and when one criterion was not met or ^(^ ^2^ ^)^ when two key criteria were adequate, but an intention-to-treat analysis was not conducted. The category ‘unclear/low risk of bias’ was assigned when two key criteria were adequately met and the rest of the criteria were adequate. Publication bias was assessed using funnel plots when more than ten studies were included in a meta-analysis^(^ ^12^ ^)^.

## Results

A total of 3069 records were retrieved and 1888 remained after deduplication. Following assessment based on title and abstract, seventy-five records were considered to be potentially relevant. Inclusion criteria were met by twenty-one RCT, but for one trial, data were not available after request (N. P. West, P. L. Horn, D. B. Pyne, V. J. Gebski, S. J. Lahtinen, P. A. Fricker and A. W. Cripps, unpublished results ‘Probiotic supplementation for respiratory and gastrointestinal illness symptoms in healthy physically active individuals’). Twenty trials were included in this systematic review (full details are provided in online Supplementary File S3): Berggren *et al.* ^(^ ^5^ ^)^; de Vrese *et al.* ^(^ ^6^ ^)^; Leyer *et al.* ^(^ ^7^ ^)^; Niborski *et al.* ^(^ ^14^ ^)^; Tiollier *et al.* ^(^ ^15^ ^)^; Tubelius *et al.* ^(^ ^16^ ^)^; Turchet *et al.* ^(^ ^17^ ^)^; Hatakka *et al.* ^(^ ^18^ ^)^ and K. Hatakka (unpublished results 2007 ‘Probiotics in the prevention of clinical manifestations of common infectious diseases in children and in the elderly’); Kumpu *et al.* ^(^ ^19^ ^)^; Cazzola *et al.* ^(^ ^20^ ^)^; Merenstein *et al.* ^(^ ^21^ ^)^; Smith *et al.* ^(^ ^22^ ^)^; Cáceres *et al.* ^(^ ^23^ ^)^; Guillemard *et al.* ^(^ ^24^ ^)^; Guillemard *et al.* ^(^ ^25^ ^)^; Prodeus *et al.* ^(^ ^26^ ^)^; Kloster *et al.* ^(^ ^27^ ^)^; Hojsak *et al.* ^(^ ^28^ ^,^ ^29^ ^)^; C. Bentley (unpublished results ‘Double blind, randomized, placebo-controlled, multicentric nutritional study to prove the efficacy and safety of a probiotic for common colds’)^(Bentley_unpublished)^. Of these trials, ten investigated the use of probiotics in children who ranged in age from 12 months to 12 years and ten were conducted in adults, of which two were conducted in elderly free-living adults. Approximately half of the trials were carried out in Western Europe: Germany; Sweden; France; Finland; Norway; Italy. The remaining trials were carried out in several different countries including the USA, Chile, Russia, Croatia and China.

The duration of probiotic treatment ranged from 3 weeks to 7 months, although the majority of trials were carried out for approximately 3 months – over the winter months. Only three trials^(^ ^14^ ^–^ ^16^ ^)^ did not report the season in which the trial was conducted, but these trials were conducted in settings such as a fireman training course, a military training course and employees working at TetraPak. Other settings included day-care centres, schools, a university, a paediatric hospital or the general population.

*Lactobacillus* strains were investigated by fifteen trials^(Bentley_unpublished,^ ^5^ ^,^ ^7^ ^,^ ^14^ ^–^ ^19^ ^,^ ^21^ ^,^ ^23^ ^,^ ^24^ ^,^ ^26^ ^,^ ^28^ ^,^ ^29^ ^)^ and strains of *Lactobacillus* administered concurrently with *Bifidobacterium* strains by five trials^(^ ^6^ ^,^ ^20^ ^,^ ^22^ ^,^ ^25^ ^,^ ^27^ ^)^. Of those trials that evaluated *Lactobacillus* strains, seven^(^ ^6^ ^,^ ^14^ ^,^ ^17^ ^,^ ^21^ ^,^ ^24^ ^–^ ^26^ ^)^ investigated the use of the fermented milk drink ‘Actimel’ (DanActive in the USA). All but one of the trials^(^ ^17^ ^)^ compared probiotics with a placebo.

Quality assessment of the studies is summarised in Table 2, and full assessments are provided in online Supplementary File S2. All the trials used appropriate randomisation methods, such as a computer-generated randomisation list or referring to a random number. Appropriate allocation concealment methods were reported in most of the studies, including the use of sealed envelopes^(^ ^7^ ^,^ ^16^ ^,^ ^18^ ^,^ ^19^ ^)^, central allocation^(^ ^6^ ^,^ ^20^ ^–^ ^22^ ^)^, and/or the use of coded packaging/containers that were identical in appearance^(^ ^5^ ^,^ ^15^ ^,^ ^21^ ^–^ ^23^ ^)^. Subjects were included sequentially in accordance with the randomisation list in four trials^(^ ^14^ ^,^ ^24^ ^–^ ^26^ ^)^. Allocation concealment was only partially addressed by two trials^(^ ^17^ ^,^ ^27^ ^)^. Among the nineteen trials^(Bentley_unpublished,^ ^5^ ^–^ ^7^ ^,^ ^14^ ^–^ ^16^ ^,^ ^18^ ^–^ ^20^ ^,^ ^22^ ^–^ ^29^ ^)^ that were described as double blind, descriptions of blinding methods were provided in most. However, due to colour coding of the study product and placebo in three trials^(^ ^18^ ^,^ ^19^ ^,^ ^27^ ^)^, it was unclear whether blinding was maintained. In this review, one open label study^(^ ^17^ ^)^ was included, so although participants were randomised, the study was not blinded.

Overall, twelve trials^(Bentley_unpublished,^ ^5^ ^,^ ^6^ ^,^ ^14^ ^,^ ^16^ ^,^ ^20^ ^,^ ^21^ ^,^ ^24^ ^–^ ^26^ ^,^ ^28^ ^,^ ^29^ ^)^ were considered to have a ‘low’ risk of bias, one^(^ ^8^ ^)^ had an ‘unclear/low’ risk of bias as all the quality criteria were well-reported except for the methods of blinding, and seven^(^ ^16^ ^–^ ^19^ ^22^ ^,^ ^23^ ^,^ ^27^ ^)^ had ‘some’ risk of bias due to imbalances in dropout rates between the treatment groups and an intention-to-treat analysis not being conducted, three studies^(^ ^18^ ^,^ ^19^ ^,^ ^27^ ^)^ due to blinding and an intention-to-treat analysis not being conducted, one study^(^ ^17^ ^)^ due to lack of blinding and imbalances in dropouts, and one study^(^ ^15^ ^)^ due to a small sample size (*n* 47). No trials had a ‘high’ risk of bias.

The included trials evaluated ‘colds’, ‘respiratory tract infections’ and ‘common infectious diseases’. The majority (*n* 17) of the trial authors reported clear descriptions of the symptoms and diagnoses of these conditions. In eleven of the trials^(^ ^15^ ^,^ ^17^ ^–^ ^20^ ^,^ ^22^ ^–^ ^25^ ^,^ ^28^ ^,^ ^29^ ^)^, a physician confirmed the presence of an infection, and in an additional two trials^(^ ^5^ ^,^ ^7^ ^)^, symptoms were reported by the participants in a diary with a diagnosis confirmed by a trial investigator.

In the included trials, three main outcomes were reported: duration of illness episodes; number of days of illness per person; absenteeism from day care/school/work. The results of studies carried out in children are reported in Table 3, of those in adults in Table 4 and of those in elderly people in Table 5.

**Table 3:** Results of included studies conducted in children (Mean values and standard deviations)

Study	Condition	Duration of treatment (months)	Treatments	Age (years)	Number randomised (included in the analysis)	Percentage of females	Number (%) of participants with at least one illness episode	Number of illness episodes	Duration of illness episodes (d)	Number of days of illness	Number of days absent
Mean	sd	Mean	sd	Mean	sd	Mean	sd
Cáceres *et al.* ^(^ ^23^ ^)^	Acute RTI	3	*L.* *rhamnosus* HN001	3·1	1·1	203 (170)	52·7	NR	172*	20·4	14·4	NR	4·70	5·5
			Placebo	3·2	1·0	195 (179)	50·8	NR	181	19·4	14·8	NR	4·05	5·6
Cazzola *et al.* ^(^ ^20^ ^)^	Common winter diseases	3	*L. helveticus* R0052, *Bifidobacterium infantis* R0033, and *Bifidobacterium bifidum* R0071+750 mg FOS†	4·1	1·0	62 (62)	46·8	29‡ (47 %)	64§	NR	NR	2·11	2·1
			Placebo	4·2	1·1	73 (50)	46·6	43 (86 %)	87§					2·88	2·8
Hatakka (2007, unpublished results) and Hatakka *et al.* ^(^ ^18^ ^)^	GI and RI	7	*L.* *rhamnosus* GG	4·6	1·5	Unclear (282)	46	97 (34 %)	NR	NR	NR∥		4·9	NR¶
			Placebo	4·4	1·5	Unclear (289)	52	123 (43 %)						5·8	NR
Hojsak *et al.* ^(^ ^28^ ^)^	Respiratory symptoms	Median: 5 d	*L. rhamnosus* GG	9·9	5·1	376 (376)	49·2	8 (2 %)	NR	NR r	8 (2·1 %)**		NR
			Placebo	10·6	5·0	366 (366)	44·3	20 (6 %)	NR	NR r	19 (5·2 %)**		NR
Hojsak *et al.* ^(^ ^29^ ^)^	Respiratory symptoms	3	*L. rhamnosus* GG	51·9 months (13–86)	139 (139)	43·9	60 (43 %)	NR	NR	39 (28·1 %)**		3·1	3·6††
			Placebo	53·6 months (13–83)	142 (142)	44·4	96 (68 %)				70 (49·3 %)**		5·1	3·6
Kloster Smerud *et al.* ^(^ ^27^ ^)^	Gastrointestinal and respiratory symptoms	7	Biola (*L. rhamnosus* GG with *L.* *acidophilus* LA-5 and *Bifidobacterium* *lactis* BB-12)	Over both groups: 18 months	117 (97)	NR	92‡ (95 %)	492	5·39	7·89‡	5·35	3·97‡	7·5	5·0††
			Placebo	Over both groups: 18 months	123 (102)	NR	95 (93 %)	564	4·69	5·19	5·94	3·77	8·5	5·0
Kumpu *et al.* ^(^ ^19^ ^)^	Respiratory symptoms	28 weeks	*L. rhamnosus* GG	4·0	1·3	261 (251)	47	239 (95 %)	NR	Median 8 d (IQR 5–12)	5·03‡‡	0·88	NR
			Placebo	4·0	1·4	262 (250)	47	236 (94 %)	NR	Median 8 d (IQR 5–12)	5·17	0·96	NR
Leyer *et al.* ^(^ ^7^ ^)^	Cold and influenza-like symptoms	6	*L. acidophilus* NCFM	3·7	0·7	110 (110)	57	NR	NR	NR	4·5	4·7	3·6	3·7‡
			*L. acidophilus* NCFM and *Bifidobacterium* *animalis* subsp. *lactis* Bi-07	3·8	0·6	112 (112)	52·7	NR	NR	NR	3·4	3·7	3·8	3·9
			Placebo	4·1	0·54	104 (104)	57·7	NR	NR	NR	6·5	7·3	5·2	5·7
Merenstein *et al.* ^(^ ^21^ ^)^	CID§§	90 d	DanActive (*L.* *paracasei* subsp. *casei* DN-114001 with *Streptococcus thermophilus* and *L.* *bulgaricus*)	4·86	1·12	314 (250 households)	50	NR	NR	NR	NR	421·5 d ‡absent (1·69 d/household)
			Placebo	4·94	1·13	324 (250 households)	46·9	NR	NR	NR	NR	463·0 d absent (1·85 d/household)
Prodeus *et al.* ^(^ ^26^ ^)^	Upper RI	3	*L. paracasei* subsp. *casei* DN-114001 with *S. thermophilus*+ *L.* *delbrueckii* subsp. *bulgaricus*	Over both groups: 4	300 (300)	Overall 45·7	66 (22 %)	98∥∥	NR	NR	5·68	4·01‡
			Placebo	Over both groups: 4	299 (299)		73 (24 %)	93∥∥	NR	NR	5·64	3·67

RTI, respiratory tract infections; NR, not reported; FOS, fructo-oligosaccharides; GI, gastrointestinal infections; RI, respiratory infections; IQR, interquartile range; CID, common infectious diseases.

* The total number of illness episodes was calculated from the total number of illness episodes per child (included in the analysis).

† Although this study used FOS along with probiotic and did not use it in the control, the level of FOS was only 750 mg, which is considered to be below an active dose^(^ ^32^ ^,^ ^33^ ^)^.

‡ Data obtained from the study authors.

§ ‘Health events’.

∥ The authors stated that the number of days with symptoms was lower in the treatment group, but the difference was not significant.

¶ To calculate mean difference and 95 % CI, standard deviation was calculated from the CI.

** Children with a RTI that lasted >3 d.

†† Standard deviation was calculated from the *P* value.

‡‡ Number of days with at least one respiratory symptom per subject per month.

§§ CID included upper RTI, lower RTI and gastrointestinal tract infections.

∥∥ Number of CID.

**Table 4:** Results of included studies conducted in adults (Mean values and standard deviations)

Study	Condition	Duration of treatment (weeks)	Treatments	Age (years)	Number randomised (included in the analysis)	Percentage of females	Number (%) of participants with at least one illness episode	Number of illness episodes	Duration of illness episodes (d)	Number of days of illness	Number of days absent
Mean	sd	Mean	sd	Mean	sd	Mean	sd
Bentley (2008, unpublished results)	Common cold	12	*Lactobacillus* *plantarum* HEAL9 (DSM 15312) and *L.* *paracasei* 8700:2 (DSM 13434)	45·3	17·1	155 (146)	Overall 66·5	72 (49 %)	87	5·6	1·3	NR	NR
			Placebo	46·6	17·2	155 (138)		76 (55 %)	98	6·7	0·8	NR	NR
Berggren *et al.* ^(^ ^5^ ^)^	Common cold	12	*L. plantarum* HEAL9 (DSM 15312) and *L. paracasei* 8700:2 (DSM 13434)	46·5	159 (137)	65·7	76 (55 %)	121	7·0	NR*	6·2	9·27†	NR
			Placebo	43·7	159 (135)	66·7	91 (67 %)	170	7·0	NR‡	8·6	10·45	NR
de Vrese *et al.* ^(^ ^6^ ^)^	Common cold	3–5·5 months	*L.* *gasseri* PA 16/8, *Bifidobacterium* *longum* SP 07/3, and *Bifidobacterium* *bifidum* MF 20/5 with *Tribion harmonis*	37	12	238 (225)	63·9	NR	158	7·0 (sem 0·5)§	NR	NR
			Placebo	38	14	241 (229)	58·9	NR	153	8·9 (sem 1·0)	NR	NR
Guillemard *et al.* ^(^ ^24^ ^)^	CID	3 months	Actimel (*L. paracasei* subsp. *casei* DN-114001+*S. thermophilus*+*L.* *delbrueckii* subsp.*bulgaricus*)	31·8	8·9	500 (500)	57	213 (43 %)	Unclear	6·9	4·5	NR	2·0	4·3∥
			Placebo	32·5	8·9	500 (500)	56	256 (51 %)	Unclear	6·5	4·5	NR	1·6	4·0
Niborski *et al.* ^(^ ^14^ ^)^	CID¶	7	*L. paracasei* subsp. *casei* DN-114001+*S. thermophilus*+*L. delbrueckii* subsp. *bulgaricus*	Overall median 21 years (range 18–29 years)	118 (118)	Overall 1·3	28 (24 %)	32	3	2·04**	3	2·29**	0·18	0·61∥
			Placebo	Overall median 21 years (range 18–29 years)	121 (121)	Overall 1·3	34 (28 %)	38	4	2·04	4	2·29	0·24	0·61
Smith *et al.* ^(^ ^22^ ^)^	Upper RTI	12 s	*L.* *rhamnosus* GG and *Bifidobacterium* *lactis* BB-12	Overall median 19 years (range 18–24 years)	114 (101)	80·2	Not clear	83 ‘cases’	NR	5·58	4·41	23 d missed work and school (0·27/‘case’)	
			Placebo	Overall median 19 years (range 18–24 years)	117 (97)	72·2	Not clear	84 ‘cases’	NR	7·11	5·07	45 d missed work and school (0·54/‘case’)	
Tiollier *et al.* ^(^ ^15^ ^)^	RTI	4	*L. paracasei* subsp. *casei* DN-114001	21·3	(0·2)	24 (24)	100 % male	11 (46 %)	17	NR	5·5	1·6	NR
			Placebo	21·3	(0·4)	23 (23)		13 (57 %)	30	NR	6·1	1·7	NR
Tubelius *et al.* ^(^ ^16^ ^)^	RTI and GI	80 d	*L.* *reuteri protectis*	44	132 (94)	35 %	NR	NR	NR	NR	Median 3 d
			Placebo	44	130 (87)	29 %	NR	NR	NR	NR	Median 3 d

NR, not reported; CID, common infectious diseases; RTI, respiratory tract infections; GI, gastrointestinal infections.

* To calculate mean difference with 95 % CI, standard deviations were imputed based on the method of de Vrese *et al.* ^(^ ^6^ ^)^.

† Standard deviations were obtained from the study authors.

‡ No *P* value reported.

§ To calculate mean difference with 95 % CI, standard deviations were calculated from the sem.

∥ Data obtained from the study authors.

¶ CID included upper RTI, lower RTI and GI.

** Standard deviation was calculated from the *P* value.

**Table 5:** Results of included studies conducted in elderly people (Mean values and standard deviations)

Study	Condition	Duration of treatment (months)	Treatments	Age (years)	Number randomised (included in the analysis)	Percentage of females	Number (%) of participants with at least one illness episode	Number of illness episodes	Duration of illness episodes (d)	Number of days of illness	Number of days absent
Mean	sd	Mean	sd	Mean	sd	Mean	sd
Guillemard *et al.* ^(^ ^25^ ^)^	Upper RTI	3	Actimel (*Lactobacillus paracasei* subsp. *casei* DN-114001+*Streptococcus thermophilus*+ *L*.*delbrueckii* subsp. *bulgaricus*)	Median 76·0	537 (535)	63·1	61 (11 %)	120	7·7	7·2	8·5	7·2	NR
			Placebo	Median 76·0	535 (535)	62·2	66 (12 %)	135	11·0	7·7	11·6	7·9	NR
Turchet *et al.* ^(^ ^17^ ^)^	CID*	3	Milk-fermented yogurt culture and *L.* *casei* DN-114001	67·1	6·0	180 (180)	Overall 67	52 (29 %)	NR	7·0	3·2	NR	NR
			No treatment	69·3	5·6	180 (180)	Overall 67	50 (28 %)	NR	8·7	3·7	NR	NR

CID, common infectious diseases; RTI, respiratory tract infections; NR, not reported.

* CID included upper RTI, lower RTI and gastrointestinal tract infections.

### Duration of illness episodes

**Fig. 1:**
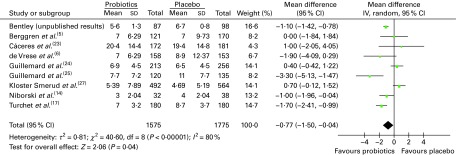
Mean duration of illness episodes (d). The ‘total’ is the overall number of illness episodes experienced by the participants (randomised in a 1:1 ratio) in each treatment group. (A colour version of this figure can be found online at http://www.journals.cambridge.org/bjn).

Among the included trials, ten^(Bentley_unpublished,^ ^5^ ^,^ ^6^ ^,^ ^14^ ^,^ ^17^ ^,^ ^19^ ^,^ ^23^ ^–^ ^25^ ^,^ ^27^ ^)^ reported on the duration of illness episodes, defined as the overall sum of illness episode lengths (in d) divided by the total number of illness episodes experienced by the study participants. Data that could be pooled in a meta-analysis were presented by nine trials^(Bentley_unpublished,^ ^5^ ^,^ ^6^ ^,^ ^14^ ^,^ ^17^ ^,^ ^19^ ^,^ ^23^ ^–^ ^25^ ^,^ ^27^ ^)^ (Fig. 1). This figure also shows the number of illness episodes in each arm of each study; the numbers are generally balanced between the arms, although slightly more illness episodes were observed in the placebo groups. Given that all the studies were randomised in a 1:1 fashion, the numbers of participants are approximately the same in each arm, and thus the numbers of illness episodes are not likely to reflect differences in sample sizes. The meta-analysis revealed that participants who received a probiotic intervention had significantly shorter illness episodes than those who received a placebo: weighted mean difference − 0·77 (95 % CI − 1·50, − 0·04), *P*= 0·04. This suggests that the mean difference in illness duration between those who take probiotics and those who do not is between half and 1 d. However, there was statistical heterogeneity among these trials (τ^2^= 0·81; *P*< 0·00 001; *I* ^2^= 80 %).

To explore potential sources of heterogeneity, subgroup analyses were conducted. Studies carried out in adults^(Bentley_unpublished,^ ^5^ ^,^ ^7^ ^,^ ^14^ ^,^ ^17^ ^,^ ^24^ ^,^ ^25^ ^)^ revealed statistical differences between the treatment groups, but heterogeneity remained. By contrast, studies carried out in children (based on only two trials^(^ ^23^ ^,^ ^27^ ^)^) did not reveal significant differences between the treatment groups. Studies conducted only in European countries (including Scandinavia) also revealed significant differences, but again, the studies were statistically heterogeneous.

**Fig. 2:**
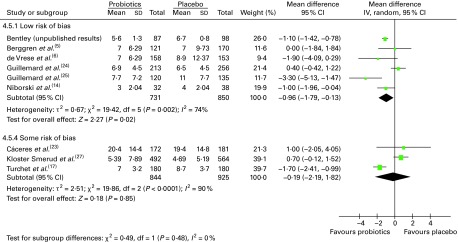
Mean duration of illness episodes (d) – analysis by risk of bias. (A colour version of this figure can be found online at http://www.journals.cambridge.org/bjn).

A subgroup analysis was also conducted by the illness as described by the study authors (i.e. acute RTI, colds and ‘common infectious diseases’) and by the duration of the trial ( < 3 months, 3–5 months, 6 months or longer), but few significant effects were observed (data not shown). Finally, an analysis of six studies considered to have a ‘low’ risk of bias yielded a significant result similar to the overall pooled analysis (weighted mean difference − 0·96 (95 % CI − 1·79, − 0·13), *P*= 0·02), but heterogeneity remained (Fig. 2).

In addition to the nine trials included in the meta-analysis, one study^(^ ^19^ ^)^ compared *Lactobacillus rhamnosus* with a placebo in 523 children aged 2–6 years attending day-care centres in Finland. The authors reported that, after 28 d of treatment, the median duration of respiratory symptom episodes was 8 d (interquartile range 5–12) in both groups (the authors did not report the number of illness episodes). However, this study had some risk of bias, so there is uncertainty regarding its results.

### Number of days of illness per person

Duration of illness per person was calculated as the overall number of days with symptoms divided by the number of individuals with an illness. In some studies, it was unclear whether all individuals were included in the analysis (i.e. participants with and without illness). To account for the possibility that different units were used to calculate this outcome, data were pooled using a SMD. We found that the numbers of people with an illness episode were either similar between the probiotic and placebo groups or slightly higher in the placebo group.

**Fig. 3:**
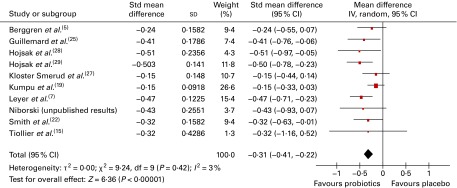
Duration of illness per person. In this meta-analysis, dichotomous and continuous data were pooled. The totals used in this analysis (not shown) were mostly the number of individuals with at least one illness episode (see Table 3). In one study^(^ ^7^ ^)^, the totals used in the analysis were the numbers of participants included in the study, and in another study^(^ ^22^ ^)^, the totals used were the numbers of ‘cases’ of illness (in both studies, the number of individuals with illness episodes was not reported). (A colour version of this figure can be found online at http://www.journals.cambridge.org/bjn).

Overall, eleven trials reported on the number of days the participants were ill, of which ten could be pooled in a meta-analysis (Fig. 3). The results demonstrated a significant difference in favour of probiotics, suggesting fewer numbers of days of illness per person compared with that in participants who had taken a placebo (SMD − 0·31 (95 % CI − 0·41, − 0·11), *P*< 0·00 001). There was no statistical heterogeneity among these studies (τ^2^= 0·00; *P*= 0·42; *I* ^2^= 3 %).

In addition to these trials, Hatakka *et al.* ^(^ ^18^ ^)^ examined the effects of *L. rhamnosus* GG *v.* placebo for 7 months in 571 healthy children aged 1–6 years from 18 day-care centres in Finland. They reported that the number of days with respiratory and gastrointestinal symptoms was lower in the *L. rhamnosus* GG group than in the placebo group, but that the difference did not reach statistical significance (no data were reported in the paper or could be obtained). They did report that the duration without respiratory symptoms was 5 weeks (95 % CI 4·1, 5·9) in the probiotic group and 4 weeks (95 % CI 3·5, 4·6) in the placebo group (*P*= 0·03). This trial had some risk of bias, so that there is uncertainty with regard to the reliability of the results.

### Absenteeism (days away from day care/school/work)

Absenteeism appears to have been largely calculated as the number of days absent from day care/school/work divided by the number of participants with at least one illness episode (i.e. absenteeism/ill person). In some trials, it was unclear how absenteeism was calculated. To account for potential differences in units, we pooled the data using a SMD.

**Fig. 4:**
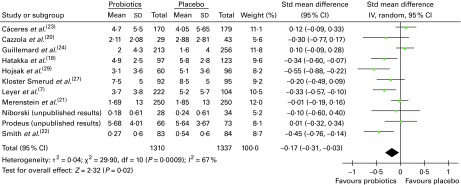
Days absent from day care/school/work. The ‘total’ is mostly the number of individuals with at least one illness episode. In two studies^(^ ^7^ ^,^ ^23^ ^)^, the totals used were the number of participants included in the study; in one study^(^ ^21^ ^)^, the totals were the number of households randomised, and in another study^(^ ^22^ ^)^, the totals were the number of ‘cases’ of illness (in all these studies, the number of individuals with illness episodes was not reported). (A colour version of this figure can be found online at http://www.journals.cambridge.org/bjn).

Overall, twelve trials reported on absenteeism due to respiratory infections/common infectious diseases, of which eleven could be pooled in a meta-analysis (Fig. 4). The results demonstrated that there was a significant difference in favour of probiotics, suggesting fewer numbers of days absent from day care/school/work compared with that in participants who had taken a placebo (SMD − 0·17 (95 % CI − 0·31, − 0·03), *P*= 0·02). However, there was statistical heterogeneity among the studies (τ^2^= 0·04; *P*= 0·0009; *I* ^2^= 67 %).

Very few subgroup differences were observed in the subgroup analyses (data not shown). A statistically significant result was obtained for eight trials conducted in children, suggesting fewer numbers of days absent from day care/school in participants who had taken probiotics than in those who had taken a placebo (SMD − 0·18 (95 % CI − 0·34, − 0·02), *P*= 0·03), although again significant heterogeneity remained. Analysis by risk of bias demonstrated no pattern. While only eleven trials were included in this analysis, the funnel plot was roughly symmetrical, indicating no publication bias (data not shown).

In the meta-analysis, one trial that evaluated absenteeism could not be included. A well-conducted trial by Tubelius *et al.* ^(^ ^16^ ^)^ examined the effects of *Lactobacillus reuteri* (ATCC55730) *v.* placebo on the length of sick leave due to respiratory or gastrointestinal infections in 262 healthy employees working at TetraPak in Sweden. They reported a median duration of 3 d sick leave in both groups (no other statistical results were reported and nor was it clear how many individuals experienced an illness episode).

## Discussion

We identified a number of studies that evaluated the effectiveness of probiotics on the duration of illness episodes. A meta-analysis of the data showed that the mean duration of illness episodes decreased by between half and 1 d in participants who received probiotics compared with those who did not. We also found significantly fewer numbers of days of illness per person and significantly fewer numbers of days absent from day care/school/work in participants who had taken probiotics than in those who had taken a placebo. However, there was significant statistical heterogeneity between the studies that reported on the duration of illness episodes and those that reported on absenteeism from day care/school/work, but not for studies that reported on the number of days of illness per person. This unexplained heterogeneity means that the effect size (i.e. the difference in the duration of illness episodes between treated and untreated individuals) may differ between the population groups.

Subgroup analyses did not elucidate sources of statistical heterogeneity among the studies. As many trials were not carried out in each subgroup, some of the analyses did not have enough power to detect any underlying effects and thus could not detect significant effects.Where differences were observed, it is important to note that inferences made according to between-study differences do not translate into within-study differences, as this would be making an indirect comparison of effects. One cannot make the assumption that the effectiveness of probiotics on the duration of illness episodes is insignificant in children compared with that in adults. Moreover, other unknown modifying factors in studies conducted in children may have had an effect on the results that could potentially be independent of age.

For two of the outcomes evaluated, we pooled the data using the SMD, which can be difficult to interpret. Based on research in the social sciences, Cohen^(^ ^30^ ^)^ suggested that effect size indices of 0·2, 0·5 and 0·8 can be used to represent small, medium and large effect sizes, respectively. Such generic interpretations may be problematic, as even small improvements may be important in health-related contexts^(^ ^12^ ^)^. We also argue that improvements in respiratory illness, even up to a day, would be of great benefit to an individual and could potentially have public health benefits.

Furthermore, two systematic reviews^(^ ^10^ ^,^ ^31^ ^)^ that evaluated the use of probiotics for the prevention of acute RTI also reported on the duration of illness. Hao *et al.* ^(^ ^10^ ^)^ reported no difference between probiotics and placebo for the mean duration of illness episodes, but they included only two trials in their meta-analysis, of which one was conducted in athletes (which were excluded from this review). However, the authors did find a difference in the incidence of RTI in favour of placebo, but this outcome has not been evaluated here. The review by Vouloumanou *et al.* ^(^ ^31^ ^)^ reported that of nine studies, three showed a significant effect in favour of probiotics and six found no difference between the probiotic and comparison groups (the authors did not conduct a meta-analysis). Again, this systematic review included athletes and children aged < 1 year, which differs from the inclusion criteria of our review. No previous systematic reviews appear to have summarised data on absenteeism from day care/school/work, and this review provides new evidence for this outcome.

The present systematic review has some limitations. First, we included trials that evaluated colds and RTI, as well as ‘common infectious diseases’, so that some of the analyses will have included patients with gastrointestinal infections. On the other hand, the significant results may be indicative of the effectiveness of probiotics in the wider population. While the majority of trial authors reported clear descriptions of the symptoms and diagnoses, a confirmed diagnosis by a physician was made in about half of the trials. It is possible that acute infections could have been under-reported or over-reported in some of these trials. As with all systematic reviews, it is possible that the addition of publications in the future could alter the results. As this review includes a large number of high-quality studies that demonstrate a consistent trend in favour of probiotics, the results are probably reliable. Further research and meta-analyses are recommended in different population groups to identify potential sources of variability in the results. It is also suggested that trial authors need to more clearly describe how their outcomes were calculated.

In conclusion, this systematic review provides evidence from a number of good-quality RCT that the average duration of respiratory illness episodes, the number of days of illness per person and the number of days absent from day care/work/school are significantly reduced with probiotic treatment compared with placebo.

## Supplementary material

To view supplementary material for this article, please visit http://dx.doi.org/10.1017/S0007114514000075
